# Multivariate Calibration and Model Integrity for Wood Chemistry Using Fourier Transform Infrared Spectroscopy

**DOI:** 10.1155/2015/429846

**Published:** 2015-10-20

**Authors:** Chengfeng Zhou, Wei Jiang, Qingzheng Cheng, Brian K. Via

**Affiliations:** ^1^Forest Products Development Center, School of Forestry and Wildlife Sciences, Auburn University, 520 Devall Drive, Auburn, AL 36849, USA; ^2^Center for Bioenergy and Bioproducts, Biosystems Engineering, Auburn University, 520 Devall Drive, Auburn, AL 36849, USA; ^3^College of Textiles, Qingdao University, 308 Ningxia Road, Qingdao 266071, China

## Abstract

This research addressed a rapid method to monitor hardwood chemical composition by applying Fourier transform infrared (FT-IR) spectroscopy, with particular interest in model performance for interpretation and prediction. Partial least squares (PLS) and principal components regression (PCR) were chosen as the primary models for comparison. Standard laboratory chemistry methods were employed on a mixed genus/species hardwood sample set to collect the original data. PLS was found to provide better predictive capability while PCR exhibited a more precise estimate of loading peaks and suggests that PCR is better for model interpretation of key underlying functional groups. Specifically, when PCR was utilized, an error in peak loading of ±15 cm^−1^ from the true mean was quantified. Application of the first derivative appeared to assist in improving both PCR and PLS loading precision. Research results identified the wavenumbers important in the prediction of extractives, lignin, cellulose, and hemicellulose and further demonstrated the utility in FT-IR for rapid monitoring of wood chemistry.

## 1. Introduction

As the most abundant fibrous material, wood is utilized in numerous areas including textile [1], paper making [2], building construction [3], composites [4], and bioenergy [5]. The chemical composition of wood including cellulose, hemicellulose, and lignin plays an important role when evaluating the utility of a feedstock for various product streams. Rapid assessment of biomass would open up opportunities for categorizing the raw material to an appropriate end use, allowing for better process control, or assist in the selection of better silvicultural or genetic management strategies for improved product performance and forest health [6]. Recently, near-infrared (NIR) spectroscopy was found to be a good tool for fast and quantitative analyses of chemistry components in plants [7–11]. NIR has been primarily successful due to its rapidity, precision, and low cost.

These same chemometric methods commonly used for NIR have been found to also be useful for Fourier transform infrared (FT-IR) spectroscopy [12]. Some researchers have studied the ability to predict secondary biomass properties with FT-IR [8, 13, 14]. Compared to NIR, FT-IR has some advantages as follows. First, not only is it advantageous for quantitative analysis, but one can also determine key functional groups associated with a particular trait of interest. Also, FT-IR spectroscopy is much more common for standard laboratory analysis of polymeric materials than NIR and is used in research institutes, laboratories, universities, and companies.

Principal components regression (PCR) and partial least squares (PLS) are the most common chemometric techniques used to construct prediction models and the loadings of the model can be further used for interpretation on contributing functional groups [15]. During woody tissue analysis, coefficients/loadings within the models are often used to interpret the relationship between wood chemistry functional groups and key traits. It was found in our previous study with NIR that PLS performed better for prediction while PCR was better for model interpretation and wavenumber selection [16]. Given that PLS optimizes both *X* and *Y* matrix for optimal prediction, it is hypothesized that PCR may also be the best for interpretation with FT-IR. Such work has not been done for the midinfrared region and is the subject of this research.

The objective of this paper was to investigate whether FT-IR coupled with chemometrics yields good predictive equations of wood chemical composition and whether the PLS modeling method introduces additional error to the loading plots.

We assumed that the loadings/coefficients in PLS modeling will decrease in precision and consequently increase in variance as a result of optimization for prediction. To test this alternative hypothesis (*H*_*a*_), it was necessary to obtain the residuals between the locations of the local peak loading (PLS and PCR) and the best representative band assignment was obtained from the literature. The mathematical expression was listed as follows:

(1) $$\begin{array}{c} W-{\text{BA}}_{L}=R, \end{array}$$

where *W* represents the wavenumber obtained through PLS or PCR analysis, BA_*L*_ is the best representative band assignment obtained from the literature, and *R* represents the residual between *W* and BA_*L*_. Then, the variance of the residuals will be further analyzed under the following hypothesis constructs:

(2) H0: σPLS-R2=σPCR-R2,Ha: σPLS-R2>σPCR-R2,

where *σ* ^2^ represents the variance of *R* obtained from PLS or PCR models. Differences for variance between model loadings will be tested by the *F*-test or *t*-test of *R* [17].

## 2. Experimental Procedures

### 2.1. Materials and Sample Preparation

Acetone (ACS Grade), sulfuric acid (98%, w/w), acetic acid (100%, w/w), sodium chlorite (High Purity Grade), and sodium hydroxide (Solution Reagent Grade, 50% w/w) were purchased from the VWR Company (Atlanta, GA, USA).

All of the hardwood samples were composed of four different genera, including 4* Eucalyptus* samples, 9 cotton wood samples, 12 aspen samples, and 12 poplar samples. These wood samples were dried in the air for two weeks and then ground to 40 mesh and 80 mesh using Wiley mill. After that, the 80-mesh samples were used for FT-IR spectra collection and the 40-mesh samples were used for wet chemistry analysis.

### 2.2. Wood Chemistry

**Figure 1:**
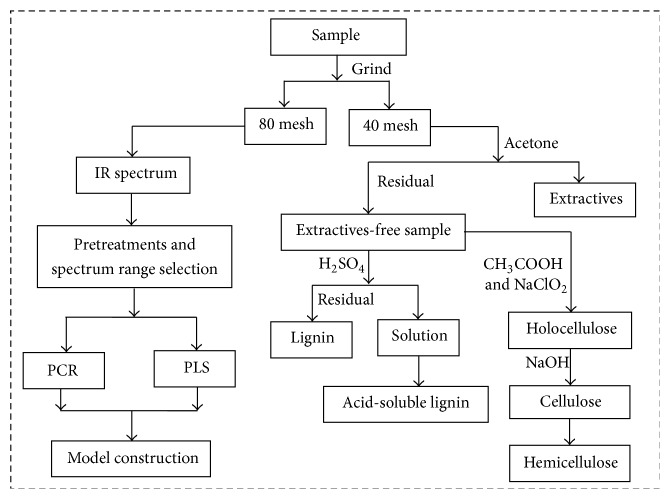
Schematic diagram for the wet chemical and FT-IR analysis process.

The extractives and lignin content were measured with National Renewable Energy Laboratory (NREL) standards [18, 19]. The cellulose and hemicellulose content were measured by traditional wet chemistry methods. The brief schematic diagram is shown in Figure 1, and the detailed processes were presented as follows.

#### 2.2.1. Extractives

One hundred fifty mL of acetone was used to extract 5 g of sample for 6 h to get acetone based extractives solution. It was then dried to remove acetone and extractives for gravimetric analysis, and the extractive-free sample was reserved for the next step.

#### 2.2.2. Lignin

A 72% (w/w) sulfuric acid treatment at 30°C for 2 h was used to prehydrolyze the extractive-free sample. The solution was then diluted to 4% sulfuric acid with distilled water, sealed in a bottle, and placed in an autoclave for 1 h at 121°C. After that, 10 mL supernatant was taken out from the bottle. Five mL supernatant was diluted to 20 mL to measure the acid-soluble lignin using ultraviolet and visible spectrophotometer (UV-Vis), and then the residual was filtered and oven dried to measure lignin content by gravimetric methods.

#### 2.2.3. Cellulose and Hemicellulose

In order to obtain the percentage of cellulose and hemicellulose, we have to calculate the holocellulose content first. We used a delignification procedure to determine the holocellulose content. First, 2 g of extractive-free samples was placed equally in two conical flasks (500 mL) with 320 mL of distilled water in each flask. Second, the flasks were placed in a water bath (75°C) and then 1 mL of acetic acid and 20 mL of 15% (w/w) sodium chlorite were added to each flask on a 1 h cycle for 4 h. After 4 h, the residues were filtered with filter paper and then oven dried for 3 h to test the holocellulose content. Then, 1.5 g of oven dried holocellulose was placed in a 250 mL conical flask. One hundred mL of 17.5% sodium hydroxide was stirred in the flask and the air was replaced with nitrogen and then the flask was immediately sealed with aluminum foil. The flask was then placed in a water bath at 20°C and stirred occasionally until the reaction was complete. The solution was then filtered through a preweighed filter paper and washed with 500 mL of distilled water. The sample was then oven dried at 105°C for 12 h and weighed. The residue was determined as cellulose and the hemicellulose content was considered to be the difference in holocellulose and cellulose.

### 2.3. FT-IR Acquisition

Oven dried samples, used for FT-IR spectra collection, were placed in a dessicator and allowed to cool to room temperature to avoid spectra fluctuation caused by rapidly changing temperatures [20]. The 80-mesh oven dried samples were placed on the diamond plate of the FT-IR machine (PerkinElmer spectrum ATR 400 FT-IR/FT-NIR spectrometer, Waltham, MA, USA) and given a pressure of 70 ± 2 psi for spectra collection. The spectra covered the range of 4,000–650 cm^−1^ at a spectral resolution of 4 cm^−1^. Each spectrum was collected from an average of 4 scans and no zero filling.

### 2.4. Chemometric Analysis

Chemometric techniques, PCR and PLS modules in Spectrum Quant + software, were used for model construction. Models were adopted using unprocessed spectra (raw) and first derivative (FD). Thirty-one samples were used to construct models and 6 samples were used for validation. Cross validation on all 37 samples was also run to ensure similar results and confirm validation results. While the populations for calibration and validation were randomly selected, the distribution of the data was checked to ensure a similar mean and range between the two populations. The coefficient of determination (*r* ^2^), root mean square error of calibration (RMSEC), and root mean square error of prediction (RMSEP) [17] were used to estimate the predictive performance of the models in this work. And the residual predictive deviation (RPD) was also measured to decide whether models were good at prediction, screening, or interpretation [21].

The principal component (PC) related to the chemical constituent during multivariate modeling was utilized for PCR coefficient/loading plots. The coefficients (*y*-axis) were connected via a smooth line in Origin software (Northampton, MA, USA) and then plotted against the wavenumbers (*x*-axis). For PLS, the regression coefficients plot was generated to represent the relationship between all of the absorbance and the specific chemical constituent. The wavenumbers were then chosen and compared to the literature.

## 3. Results and Discussion

### 3.1. Assessment of Loading Plots

**Figure 2:**
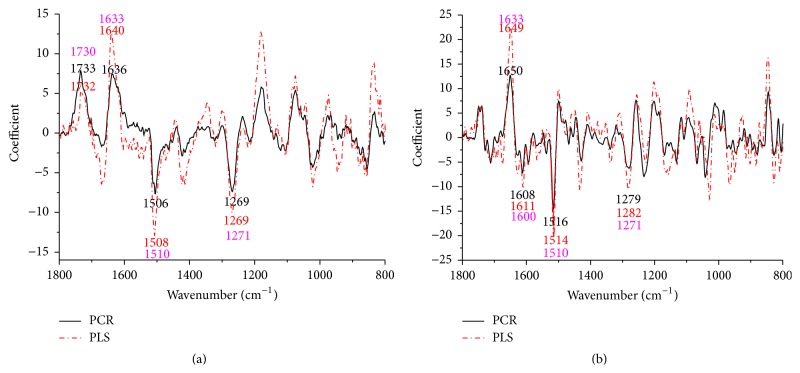
Coefficients by wavenumber for PCR and PLS for extractives prediction (a) when raw spectra were processed and (b) when a first-derivative pretreatment was processed. PC numbers 9, 2, 3, and 1 were chosen for PCR-raw, PLS-raw, PCR-derivative, and PLS derivative, respectively (*α* = 0.05).

**Figure 3:**
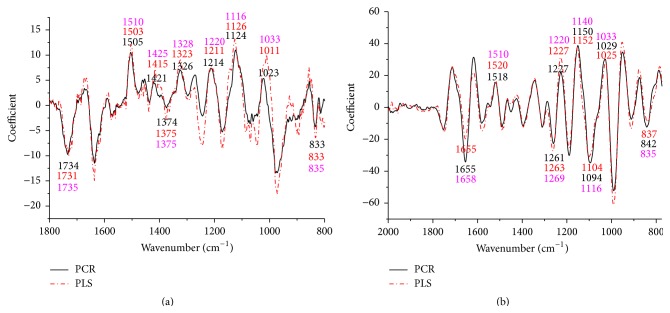
Coefficients by wavenumber for PCR and PLS for lignin prediction (a) when raw spectra were processed and (b) when a first-derivative pretreatment was processed. PC numbers 6, 2, 2, and 1 were chosen for PCR-raw, PLS-raw, PCR-derivative, and PLS derivative, respectively (*α* = 0.05).

**Figure 4:**
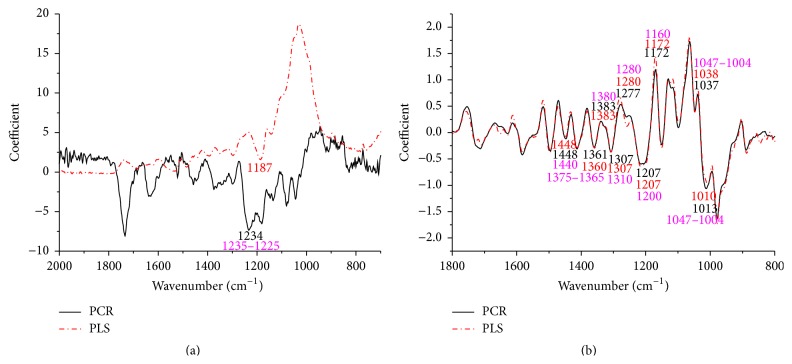
Coefficients by wavenumber for PCR and PLS for cellulose prediction (a) when raw spectra were processed and (b) when a first-derivative pretreatment was processed. PC numbers 8, 3, 1, and 2 were chosen for PCR-raw, PLS-raw, PCR-derivative, and PLS derivative, respectively (*α* = 0.05).

**Figure 5:**
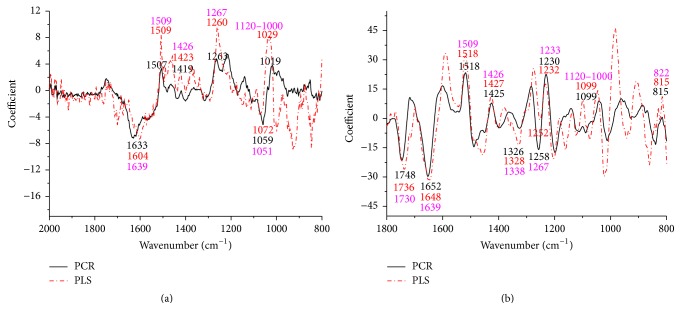
Coefficients by wavenumber for PCR and PLS for hemicellulose prediction (a) when raw spectra were processed and (b) when a first-derivative pretreatment was processed. PC numbers 3, 3, 1, and 1 were chosen for PCR-raw, PLS-raw, PCR-derivative, and PLS derivative, respectively (*α* = 0.05).

The coefficients/loadings within the models are effective ways to interpret the relationship between wood chemistry functional groups and corresponding wood chemistry percent. For extractives, lignin, and cellulose loading plots, application of the first-derivative pretreatment was made prior to PLS and PCR execution. This was found to improve loading plots as evidenced by more parallel patterns (Figures 2, 3, and 4). The positive effect of the first-derivative pretreatment within the PLS coefficient plots was probably ascribed to the removal of the baseline shift present in the raw spectra. However, the positive effect of the first-derivative pretreatment was not apparent during the prediction of hemicellulose (Figure 5). This suggests that, during model development, application of the first derivative can generally improve the precision of the loadings, but not always.

It was also apparent that there was a shift in the location of the loading between native and first-derivative based data sets. For example, the wavenumber at 1640 cm^−1^, 1508 cm^−1^, and 1269 cm^−1^ shifted to 1649 cm^−1^, 1514 cm^−1^, and 1282 cm^−1^ for the extractive calibration models when the first-derivative pretreatment was used. This loading shift at 15 to 35 cm^−1^ could also be seen for lignin (1211 to 1227 cm^−1^), cellulose (1187 to 1207 cm^−1^), and hemicellulose (1633 to 1652 cm^−1^) (Figures 3, 4, and 5). These shifts will be studied statistically in Table 1.

### 3.2. Wavenumber Assignments

Important wavenumbers for the prediction of extractives, lignin, cellulose, and hemicellulose were identified through the loading plots in Figures 2, 3, 4, and 5, and the functional groups important for a given wavenumber were further confirmed by the literature.

#### 3.2.1. Extractives

The sensitivity of the bands at 1730 cm^−1^ was attributable to the C=O stretching vibrations produced by the ester carbonyl. These peaks appear when the lipophilic fraction of extractive is studied; they may come from fat, wax compounds or in esterified resin acids [22–25]. The peak near 1600 cm^−1^ can be assigned to either the C=C stretching or an aromatic ring deformation mode [26]. The strong peak at 1510 cm^−1^ is assigned to the deformation vibration within benzene rings [22, 27]. This peak is characteristic of aromatic compounds in wood and wood extractives [28, 29]. The weak olefinic double bond stretching is exhibited at 1633 cm^−1^ [26]. The strong band at 1271 cm^−1^ was due to carbon single bonded oxygen but is more likely to be an interaction band between carbon single bonded oxygen stretch and in-plane carbon single bonded hydroxyl bending in carboxylic acids, which is usually masked by a methylene scissoring band due to the methylene group attached to the carbonyl [22, 24].

#### 3.2.2. Lignin

Coefficients by wavenumber for PCR and PLS for lignin prediction are displayed in Figure 3. C=O stretching and aromatic skeletal vibration were important loadings at 1735, 1658, 1328, 1510, and 1425 cm^−1^, respectively [30–33]. Phenolic OH and aliphatic C-H in methyl groups were important based on the loading at 1375 cm^−1^. The G ring showing with carbonyl stretching was a key functional group that was important based on the loading at 1269 cm^−1^. The C-C, C-O, and C=O stretch were important due to the variation in the loading at 1220 cm^−1^. The C-H in-plane deformation of the G ring plus secondary alcohols and C=O stretch were evident based on the loading at 1140 cm^−1^. Aromatic C-H deformation in the S ring was based on the loading at 1116 cm^−1^ [30, 34]. The aromatic C-H in-plane deformation plus C-O deformation in primary alcohols plus the C=O stretch was based on the loading at 1033 cm^−1^. C-H out-of-plane deformation in positions 2 and 6 of S rings was based on the loading at 835 cm^−1^ [30–32].

#### 3.2.3. Cellulose

As shown in Figure 4, the wavenumbers demonstrate that the O-H in-plane bending (1440 and 1220 cm^−1^), C-H bending (1380, 1375–1365, and 1280 cm^−1^), and CH_2_ wagging at 1310 cm^−1^ were important in the prediction of cellulose. The C-O-C asymmetric stretching, assigned to cellulose, appears at 1160 cm^−1^ and the C-O stretch in cellulose occurred at 1047–1004 cm^−1^ [35, 36].

#### 3.2.4. Hemicellulose

The loadings corresponding to FT-IR spectra of the hemicelluloses are shown in Figure 5(a). The peak multiplicity between 1120 and 1000 cm^−1^ is a typical characteristic of carbohydrates. The signal at 1051 and 1008 cm^−1^ corresponding to the glycosidic (C-O-C) stretching cannot be clearly distinguished due to the multiplicity of the peaks in that region. Signals at 1453, 1426, and 1338 cm^−1^ are attributed to -CH_2_ symmetric bending, CH and OH bending, and -CH wagging, respectively [37]. The signal at 1639 cm^−1^ is due to the absorbed water, but it can also reveal the presence of conjugated carbonyl groups that either are present in the polyphenolic structure of lignin or exist in uronic acids or result from carbohydrate oxidation and acetylated residues [38]. Hardwood xylan is heavily O-acetylated; therefore, the absorption of carbonyl groups is mainly contributed by xylan in hemicellulose. The characteristic peak at 1509 cm^−1^ which indicates the aromatic skeletal vibration of lignin and, correspondingly, the band at 1267 cm^−1^ associated with guaiacyl nuclei and the band at 1233 cm^−1^ related to syringyl nuclei of plane at positions 2, 5, and 6 in G units could not be distinguished. The peak at 822 cm^−1^ was attributed to C-H out of plane at positions 2 and 6 of S units and all positions of H units were not detected [39].

### 3.3. Interpretation of Significant Coefficients and Loadings Error Assessment

As we discussed in Section 3.2, C=O and C=C were key functional groups that were based on the loadings at 1730 and 1600 cm^−1^ for the prediction of extractives. For lignin prediction, the important loadings of OH and CH appeared at 1375 cm^−1^, and C-C, C-O, and C=O loadings were assigned at 1220 cm^−1^. For cellulose prediction, the C-H and CH_2_ bond was important based on loadings at 1380, 1280, and 1310 cm^−1^. Hemicellulose yielded -CH_2_, CH, and OH bending and -CH wagging bond corresponding to the loadings at 1453, 1426, and 1338 cm^−1^.

**Figure 6:**
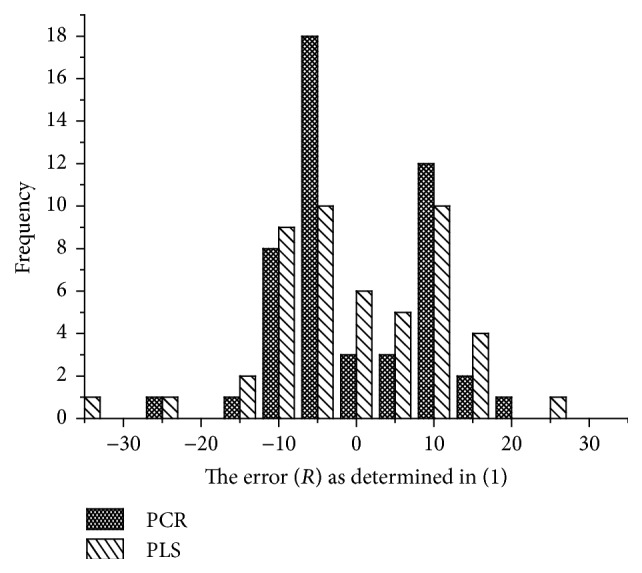
Frequency of *R* from PLS and PCR loadings of wood chemistry models.

From this study, it is apparent that error in band assignments exists and can be quantified as BA_*L*_. The distribution of error (*R*) for PCR exhibited a distribution closer to normality than PLS. Furthermore, PCR demonstrated a higher frequency of lower error in the loading estimates which supports the hypothesis that PCR provides better precision in wavenumber assignment (Figure 6). Finally, both PLS and PCR (*α* = 0.05) overlapped with zero when a confidence interval test was performed (Table 1), which is indicative of lack in bias for either PLS or PCR.

Hypothesis testing was used to test the precision of peak loading location. The statistical results of the *F*-test indicated that the variance of *R* for PLS was greater than that of PCR. This means that the alternative hypothesis (*H*_*a*_) was correct and that PCR is a better tool for assignment or interpretation of wavelengths and corresponding functional groups through multivariate modeling. Thus, PLS proved to be a better multivariate tool for prediction while PCR was better for interpretation. This was supported by another study in which two-dimensional correlation analysis and waterfall plots were used for detecting positional fluctuations of spectral changes. It was shown that 2D correlation analysis of the spectra was clear in defining the very characteristic cluster pattern both for the band position shifts and for partitioning out two overlapped bands. Their study also found principal components analysis (PCA) to be very sensitive to peak shifts and helped to justify PCR for shift identification [40]. Practically speaking, this study found that the error (±2 standard deviations in peak loadings, Table 1) for PCR loadings was approximately ±15 cm^−1^. This suggests that when loadings from PCR plots are used for interpretation, one can expect this estimate to be ±15 cm^−1^ of the true value.

In this study, transforming the spectra with the first derivative was found to improve loading plot precision. It is perhaps even possible to use PLS with competitive performance in loading precision although this study found PCR to still perform better.

### 3.4. Predictive Diagnostics

**Figure 7:**
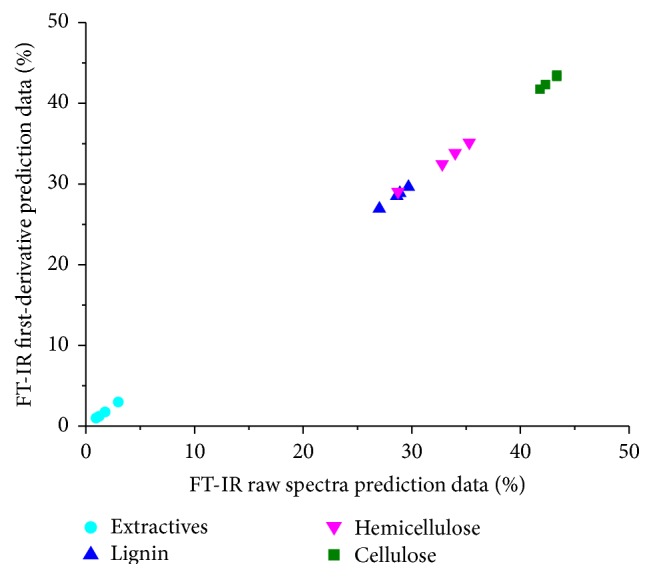
Chemical content (%, w/w) predicted by multivariate models with FT-IR (PLS) for validation samples.

The predictive results for the chemical composition in wood were shown in Table 2. It should be noticed that the assignment of the peaks is a very important reference for modeling optimization. During the model construction, coupled with the peak assignment in the previous section, the optimal wavenumber ranges of extractives, lignin, cellulose, and hemicellulose for building the FT-IR model were found to be 1750–1250 cm^−1^, 1800–800 cm^−1^, 1500–1000 cm^−1^, and 1750–800 cm^−1^, separately. It was found that PLS always outperformed PCR in predictive diagnostics with the first-derivative pretreatment often improving calibration statistics. Models with a higher *r* ^2^, a lower RMSEP, and a higher RPD were deemed to generally be the best performers. RPD has particularly been utilized as a way of classifying whether a model was used for screening, prediction, or actual measurement [7]. The best predictive results (RPD value) for extractives, lignin, cellulose, and hemicellulose were 4.18, 4.83, 1.72, and 2.04, which were performed by PLS and first-derivative pretreatment. The RPD values demonstrate that the FT-IR prediction models for extractives and lignin were good enough to conduct quantitative analysis, while cellulose and hemicellulose models were more appropriate for screening or for measurement of population statistics during process control [41]. The predictive ability of calibration models on new samples is presented in Figure 7. Several chemical components could be predicted from a single measurement in a rapid and precise way.

## 4. Conclusion

PLS and PCR were paired with FT-IR spectroscopy to determine which methods were better for interpretation and prediction of wood chemistry. Important wavenumbers to extractives, lignin, cellulose, and hemicellulose were identified by analyzing the loading plots and this was compared to the literature. It was found that PCR performed better than PLS for interpretation when FT-IR spectroscopy was used. Research also found that the chemical composition content in wood could be predicted by FT-IR models with extractives and lignin models exhibiting an RPD > 4, which suggests that these models are adequate for quantitative analysis of individual samples.

## Figures and Tables

**Table:** (a) *t*-test

	PCR	PLS
Mean *R*	0.06	−0.49
Variance	55.5	92.6
Standard deviation	7.45	9.62
95% CI	0.06 ± 2.1	−0.49 ± 2.8
Observations	49	49
Degrees of freedom	48	48
*F* value	1.67	
*P* for *t*-test	0.0796^*∗*^	

**Table:** (b) *F*-test

	PCR	PLS
Mean *R*	0.06	−0.49
Variance	55.5	92.6
Standard deviation	7.45	9.62
95% CI	0.06 ± 2.1	−0.49 ± 2.8
Observations	49	49
Degrees of freedom	48	48
*F* value	0.60	
*P* (*F* < *f*) one tail	0.0398^*∗*^	
*F* critical one tail	0.62	

^*∗*^This means that the *t*-test and *F*-test were significant with 95% confidence.

**Table 2:** Calibration and predictive results of FT-IR based multivariate models.

Algorithm	Chemical components	Wavenumber ranges	Raw spectra			First derivative		
			*R* ^2^	RMSEP	RPD	*R* ^2^	RMSEP	RPD
PLS	Extractives	1750–1250 cm^−1^	73.51	1.19	1.19	86.66	0.34	4.18
	Lignin	1800–800 cm^−1^	87.76	1.05	2.30	90.06	0.50	4.83
	Cellulose	1500–1000 cm^−1^	56.74	1.38	1.53	85.62	0.80	1.72
	Hemicellulose	1750–800 cm^−1^	79.31	2.25	1.73	92.90	1.90	2.04

PCR	Extractives	1750–1250 cm^−1^	57.93	0.87	1.69	71.04	0.59	2.41
	Lignin	1800–800 cm^−1^	73.70	1.51	1.60	87.36	0.82	2.94
	Cellulose	1500–1000 cm^−1^	52.61	1.59	1.32	48.15	0.94	1.34
	Hemicellulose	1750–800 cm^−1^	30.09	4.14	0.94	52.69	3.34	1.16
